# Consumers’ knowledge and experiences of adverse drug reaction reporting in Australia: a national survey

**DOI:** 10.1007/s00228-024-03729-y

**Published:** 2024-07-12

**Authors:** Mohammed Gebre Dedefo, Renly Lim, Gizat M. Kassie, Elizabeth Roughead, Lisa Kalisch Ellett

**Affiliations:** Quality Use of Medicines and Pharmacy Research Centre, UniSA Clinical and Health Sciences, University of South Australia, Adelaide, Australia

**Keywords:** Adverse drug reaction, Drug safety, Digital technology, Pharmacovigilance, Adverse drug reaction reporting systems, Consumer

## Abstract

**Abstract:**

This study aimed to investigate the current knowledge and experiences of consumers in Australia on adverse drug reaction (ADR) reporting and their reasons for reporting or not reporting ADRs, with a focus on the use of digital tools for ADR reporting.

**Methods:**

A cross-sectional online survey was conducted among adults who had taken medicine in Australia. A structured questionnaire with multiple choice or Likert scale responses with an option for participants to provide free-text responses and pretested for face validity was used. Consumer characteristics, knowledge, and ADR reporting practices were analyzed using descriptive statistics and the chi-square test or Fisher’s exact test.

**Results:**

A total of 544 survey responses were included in the analysis. The majority of respondents were women (68%), and 22% were aged between 65 and 74 years. Fifty-eight percent (*n* = 317) of respondents knew that they could report ADRs to either the Therapeutic Goods Administration (TGA), state or territory government health department, or healthcare professionals. Three-quarters (*n* = 405) of respondents stated that they had experienced an ADR; of these, 36% reported an ADR to either the TGA, state or territory government health department, or healthcare professionals. Among those who reported ADRs, 58% were unaware that they could use digital tools to report ADRs. The main reason for not reporting was that they did not think the ADR was serious enough to report (39%).

**Conclusion:**

Over half of consumers knew that they could report ADR; however, improved consumer awareness about using digital tools for ADR reporting and increased ADR reporting is needed.

**Supplementary Information:**

The online version contains supplementary material available at 10.1007/s00228-024-03729-y.

## Introduction

Adverse drug reactions (ADRs) are a major cause of mortality and morbidity in health care [1, 2]. In Australia, up to 3% of all hospital admissions are due to medication-related harm, resulting in an estimated 250,000 admissions and AUD$ 1.4 billion in costs annually [3, 4]. Worldwide, ADRs are associated with 5 to 17% of all hospital admissions [5–8]. ADRs are associated with an estimated 106,000 deaths annually in the United States of America (USA) [5] and are associated with an estimated 197,000 deaths annually in Europe [6]. Strengthening the pharmacovigilance system is important to monitor the safety of medicines when used in real-world settings, to reduce the burden of ADRs, and to prevent people from experiencing avoidable harm due to ADRs from medicines.

Pharmacovigilance is defined as the “science and activities relating to the detection, assessment, understanding, and prevention of adverse effects or any other medicine-related problem” [9]. Adverse event reporting is one method of post-market pharmacovigilance of medicines and anyone — healthcare professionals (HCPs), sponsors of medicine (pharmaceutical companies), and consumers can report suspected ADRs. Pharmaceutical companies are mandated to report ADRs in many countries [10–12]. The reporting of ADRs by consumers has been shown to provide early detection of known ADRs [13] and unknown adverse effects of medicines [13], add useful information on the impact of ADRs on quality of life [14], and improve the involvement of patients in the management of their health [13–15].

International studies have shown that the ADR reporting rate by consumers is low due to limited awareness of ADR reporting systems, misunderstanding about who should report the ADR, and difficulties with reporting procedures [16–18]. For example, studies conducted in the UK, Malaysia, India, South Korea, and Saudi Arabia found that 4 to 15% of consumers know about the ADR reporting systems in their countries [17–22]. After becoming aware of the ADR reporting mechanisms, 59% of Korean consumers expressed an interest in self-reporting ADRs, indicating that lack of awareness rather than lack of motivation may be a factor in the underreporting of ADRs by consumers [18]. Similar results were seen in a survey conducted in 2012 in Australia, which found that 10.4% of consumers were aware of the ADR reporting scheme for consumers in Australia [23]. In the 2012 Australian study, 21% of respondents had reported an ADR, and the majority had reported the ADR via the telephone [23].

In the 11 years, since the previous Australian study was published, the use of digital tools (e.g., mobile phones, tablets, and computers) for health-related activities has become more common. International studies have indicated that the use of digital tools can improve the reporting of adverse events by patients and healthcare professionals [24–26]. A meta-analysis that included seven studies found that the use of digital tools for ADR reporting doubled the rate of reporting compared to traditional reporting methods [24]. It is currently unknown how consumers in Australia utilize digital tools for reporting ADRs, as well as the motivations behind their decision to report or not report ADRs.

Therefore, the aim of this study was to investigate the current knowledge and practice of consumers in Australia on ADR reporting, including consumers’ reasons for reporting or not reporting ADRs, with a focus on the use of digital tools for ADR reporting.

## Methods

### Study design

A cross-sectional online survey was conducted using Qualtrics [27]. Adults aged 18 years or over living in Australia who self-reported that they had taken a medicine in the past 6 months were eligible to participate.

### Recruitment strategy

A convenience sampling technique was used to recruit participants. A flyer containing information on the study’s purpose with a quick response (QR) code and survey link allowing participants to access the survey was prepared. Various methods were used to distribute the survey flyer. The prepared survey flyer was advertised on social media (including Twitter and Facebook). Additionally, the survey flyer was displayed in community pharmacies, hospital pharmacies, and general practitioner (GP) clinic waiting areas. The flyers were also distributed to community pharmacies with the assistance of the Pharmaceutical Society of Australia (PSA).

### Sample size and data collection

We estimated that a minimum survey response sample size of 173 consumers was required in order to ensure the representativeness of the general population in our survey. The sample size was estimated using a single population proportion formula based on the prevalence of medication use in Australian adults taking one or more medicines (87.1% of Australian adults) [28], the proportion of consumers that were aware of ADR reporting in Australia (10.4%) [23], a confidence level of 95% and a 5% margin of error, and assuming a 20% non-response rate. The data collection period was from May to September 2023.

### Questionnaire development

Previous literature on the topic [23, 25, 26, 29–32] was reviewed to develop a structured questionnaire to address our research questions. The survey contained 27 items. Items in the questionnaire were categorized into five sections: (i) general questions on the participant’s experience with medicine use (four items), including the type of medicine taken, average number of medicines taken, and whether they had started taking a new medicine; (ii) knowledge of ADR reporting (six items), including knowledge that consumers can report, knowledge of where to report, knowledge of how to report, knowledge of reporting methods, awareness of the blue card adverse reaction reporting form and the online TGA form, and awareness of the black triangle scheme; (iii) practices of ADR reporting (six items), including whether they had ever experienced an ADR, whether they had reported an ADR, reason for not reporting an ADR, reason for reporting an ADR, to whom they reported, and how they reported; (iv) views on the use of digital tools for ADR reporting (seven items), including the participant’s level of experience with smartphones, their use of digital tools for healthcare activities, their use of digital tools for ADR reporting, the reasons for not using digital tools for reporting ADR, their perception of the importance of various features of digital tools, their intention to use digital tools, and their preferred method of reporting ADRs; and (v) consumers’ socio-demographic characteristics (four items), including gender, age, level of education, and state of residence. Three items from the knowledge section, six items from the practice section, and one item containing eight lists of consumers’s views on the importance of various features of digital tools were adapted from the previous literature [23, 25, 26, 29–31] and one item containing nine lists of consumers views on their intention to use digital tools to report ADRs was developed based on the combined technology of acceptance model and theory of planned behavior (C-TAM-TPB) [32]. The remaining items were developed by the authors to address the research question. The term “digital tool” was defined in the questionnaire as a mobile app, web app, or website that generates data and allows users to create a medicine side effect report on a portable device such as a smartphone, tablet, or laptop. The questions required multiple choice or Likert scale responses with the option for the participant to provide additional comments as free text responses. The full survey questionnaire is available in Supplementary material 1. In this paper, we report consumers’ socio-demographics, medicine use experience, knowledge and practice of ADR reporting, and experience with digital tool use components of the survey. Prior to dissemination, the questionnaire was pilot-tested by six people for face validity. Following the pilot test, we made minor edits to one of the questions to improve its clarity for consumers; we rearranged the order of the list of options in one of the questions and we also reshuffled the order of two questions. The time required to complete the survey was approximately 15 min.

### Data analysis

Responses from participants who completed at least the first two sections of the questionnaire relating to experiences with medicine use and knowledge of ADR reporting were included in the analysis. In total, 592 participants responded to the survey, and out of these, 544 responses met the predefined criteria and were included in the analysis. For those who fulfilled the abovementioned criteria but had some missing data, the missing information was recorded as “no response.”

Descriptive statistics were used to describe the characteristics of the survey participants. Consumers who had experienced ADRs were categorized as those who had reported or not reported ADRs, and the chi-square test was conducted to determine whether there were statistically significant differences between gender, age, level of education, average number of medicines taken, use of digital tools for healthcare activities, level of experience with smartphones, awareness of the blue card adverse reaction reporting form, awareness of the black triangle scheme, and whether a consumer had or had not reported ADRs. Fisher’s exact test was used when more than 20% of the cells had expected frequencies less than 5. Statistical Package for Social Sciences (SPSS) version 28 was used for data analysis. A *p*-value less than 0.05 was considered statistically significant. We conducted a post hoc analysis for variables with *p* < 0.05 in the chi-square test for multiple comparisons using Bonferroni correction. An adjusted *p* < 0.008 was considered statistically significant.

## Results

### Sociodemographic characteristics

A total of 544 survey responses were included in the analysis. The majority of respondents were women (68%), and 22% were aged between 65 and 74 years. The majority of respondents had a high level of education, with 32% of respondents having a bachelor’s degree and 31% having a postgraduate degree as their highest level of education. Almost all (92%) of the respondents took medicines prescribed by healthcare professionals. Fifty-six percent of respondents took one to four regular medicines, and one-third took five or more medicines. Almost half (48%) of the survey respondents had started taking a new medicine in the past 6 months (Table 1).

**Table 1:** Characteristics of the respondents and their experience with medicine use (*N* = 544)

**Characteristics of the respondents**		**Number (%)**
Gender	Man	113 (20.8%)
	Woman	370 (68.0%)
	Other gender or no response	61 (11.2%)
Age	18–24	29 (5.3%)
	25–34	70 (12.9%)
	35–44	61 (11.2%)
	45–54	91 (16.7%)
	55–64	89 (16.4%)
	65–74	118 (21.7%)
	75 or older	35 (6.4%)
	No response	51 (9.4%)
Highest level of education	Secondary school	88 (16.2%)
	VET certificate, diploma, associate diploma, associate degree or graduate diploma	36 (6.6%)
	Bachelor's degree	176 (32.4%)
	Postgraduate degree	170 (31.3%)
	Others	8 (1.5%)
	No response	66 (12.1%)
State of residence	Victoria	155 (28.5%)
	New South Wales	119 (21.9%)
	Queensland	94 (17.3%)
	South Australia	84 (15.4%)
	Western Australia	9 (1.7%)
	Tasmania	9 (1.7%)
	The Australian Capital Territory	7 (1.3%)
	Northern Territory	5 (0.9%)
	No response	62 (11.4%)
Type of medicine taken in the past 6 months^a^	Medicines prescribed by a healthcare professional	500 (91.9%)
	Non-prescription or over-the-counter medicines	348 (64.7%)
The average number of medicines taken in the past 6 months	1–4	305 (56.1%)
	≥ 5	186 (34.2%)
	No response	53 (9.7%)
Did you start taking a new medicine for the first time in the past 6 months?	Yes	259 (47.6%)
	No	281 (51.7%)
	Not sure	4 (0.7%)

*VET* Vocational education and training

^a^Respondents had the option to choose multiple responses

### Knowledge of consumers on ADR reporting

Fifty-eight percent of respondents (*n* = 317) indicated that they knew they could report ADRs (Table 2). Of the 317 respondents who knew that they could report ADRs, 81% knew that they could report ADRs to healthcare professionals and 62% knew that they could report ADRs to Australia’s national medicines regulator, the Therapeutic Goods Administration (TGA). Seven percent of respondents did not know where to report ADRs, even though they knew that they could report them (Table 2). Thirty-five percent of respondents (*n* = 190) knew how to go about reporting an ADR. Online reporting via the TGA website (66% of the 190 respondents who knew how to report an ADR) was the most commonly known method for ADR reporting by participants. The blue card adverse reaction reporting form was not well known by participants, with 82% responding that they had not heard of this before. Similarly, 85% of respondents had not heard about the TGA black triangle scheme (Table 2).

**Table 2:** Respondents’ knowledge of ADR reporting

**Questions**	**Responses**	**Number (%)**
1. Did you know that you can report side effects to the Therapeutic Goods Administration, State or territory government health departments, or healthcare professionals? (*N* = 544)	No	227 (41.7%)
	Yes	317 (58.3%)
1.1. Please select the organisations you are aware of where side effects can be reported to.^ab^ (*N* = 317)	Healthcare professionals	258 (81.4%)
	The Therapeutic Goods Administration	197 (62.1%)
	The State or Territory government health department	130 (41.0%)
	I am not sure where I can report to	23 (7.2%)
	Pharmaceutical company	6 (1.9%)
2. If you wanted to report a side effect due to a medicine to the Therapeutic Goods Administration, state or territory government health department or healthcare professionals, would you know how to go about it? (*N* = 544)	No	236 (43.4%)
	Not sure/no response	118 (21.7%)
	Yes	190 (34.9%)
2.1. Which side effect reporting method(s) are you aware of?^ab^ (*N* = 190)	Online via the TGA website	125 (65.8%)
	Phone call	102 (53.7%)
	Email	90 (47.4%)
	Post	26 (13.7%)
	Others	15 (7.9%)
3. Have you heard about the blue card adverse reaction reporting form? (*N* = 544)	Yes	73 (13.4%)
	No	448 (82.4%)
	Not sure/no response	23 (4.2%)
4. Have you heard about the Black Triangle scheme? (*N* = 544)	Yes	58 (10.7%)
	No	460 (84.6%)
	Not sure/no response	26 (4.8%)

^a^This question was only presented to respondents who answered “Yes” to the previous question

^b^Respondents had the option to choose multiple answers

### Practices of consumers on ADR reporting and their experience of using digital tools

Three quarters of respondents (*n* = 405) stated that they had experienced an ADR; of these, 36% (*n* = 145) had reported an ADR. The 145 respondents who had reported an ADR were asked to describe which individual or organizations they had reported their ADR. The majority of the respondents had reported their ADR to a healthcare professional (80%) and mainly reported their ADR in person (69%). When asked about their use of smartphones, 71% of respondents indicated that they used their smartphones for activities such as making e-payments and booking appointments using apps. Almost all (90%) of the respondents had used digital tools for healthcare activities, including making or confirming doctor’s appointments. Of the 145 respondents who stated that they had reported ADRs, 83% responded that they had not used a digital tool for reporting ADRs. The major reason the respondents did not use a digital tool to report the ADR was that they were not aware that they could report using digital tools (58%) (Table 3). Twenty respondents provided reasons for not using digital tools to report ADRs in the free-text field. Commonly reported reasons were that they preferred to discuss their ADRs with their healthcare professionals in person (*n* = 11) and that they had experienced the ADR before the era of digital tools (*n* = 3).

**Table 3:** Respondents’ practices of ADR reporting and their experience of using digital tools

**Questions**	**Responses**	**Number (%)**
1. Have you ever experienced a side effect from any medicine? (N = 544)	No	95 (17.5%)
	Not sure/no response	44 (8.1%)
	Yes	405 (74.4%)
1.1. Have you ever reported a medicine side effect to the TGA, state or territory government health department, or healthcare professionals? (*N* = 405)	No	254 (62.7%)
	Not sure	6 (1.5%)
	Yes	145 (35.8%)
1.1.1. Who did you report the medicine’s side effect (s) to?^ac^ (*N* = 145)	Healthcare professional	116 (80.0%)
	TGA	22 (15.2%)
	The State or Territory health department	14 (9.6%)
	Others	3 (2.1%)
1.1.2. How did you report the most recent side effect you experienced?^ac^ (*N* = 145)	In-person	100 (68.9%)
	Via Phone conversation	34 (23.4%)
	Online form via the TGA website	17 (11.7%)
	Via Email	9 (6.2%)
	Via Post	8 (5.5%)
	Others	1 (1.4%)
1.1.3. Have you ever used digital tools for reporting medicine side effects? (*N* = 145)^a^	Yes	21 (14.5%)
	Not sure/no response	4 (2.7%)
	No	120 (82.8%)
1.1.3.1. Why didn’t you use a digital tool for reporting the side effects?^bc^ (*N* = 120)	I wasn’t aware that I could report using digital tools	70 (58.3%)
	I have data privacy concerns	16 (13.3%)
	I find online reporting confusing	11 (9.2%)
	I have security concerns	10 (8.3%)
	I didn’t have an electronic device	4 (3.3%)
	Other reasons	20 (16.7%)
2. How would you rate your level of experience with using a smartphone? (*N* = 544)	Limited (I use it to make a phone call and send text messages only)	21 (3.9%)
	Moderate (I use it for online searches such as on Google and Safari)	102 (18.8%)
	Advanced (I use it to make e-payments, book appointments using apps etc.)	388 (71.3%)
	I don’t use a smartphone	3 (0.6%)
	No response	30 (5.5%)
3. Have you ever used digital tools for healthcare activities, for example, making or confirming doctor’s appointments? (*N* = 544)	Yes	490 (90.1%)
	No	33 (6.1%)
	Not sure/no response	21 (3.9%)

*TGA* Therapeutic Goods Administration

^a^This question was only presented to respondents who answered “Yes” to the previous question

^b^This question was only presented to respondents who answered “No” to the previous question

^c^Respondents had the option to choose multiple answers

### Consumers’ reasons for reporting ADRs

**Fig. 1:**
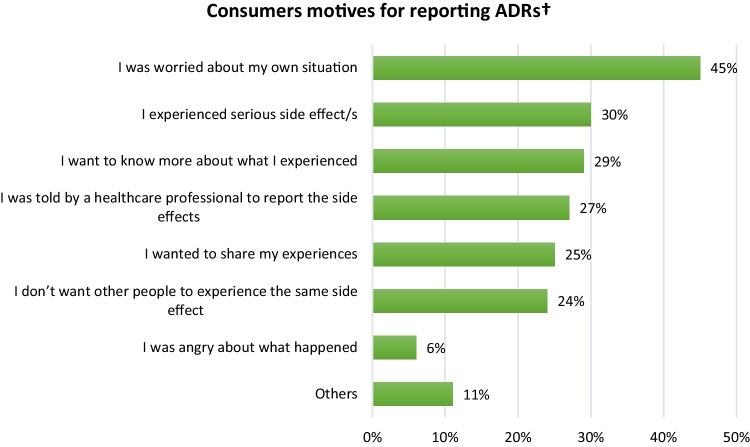
Motives of consumers who experienced ADRs and reported them (*N* = 145). †Respondents had the option to choose multiple responses

The 145 respondents who had reported an ADR were asked why they decided to report the ADR. Common reasons for reporting ADRs were that they were worried about their own situation (45%), and they experienced serious ADRs (30%) (Fig. 1). Respondents were asked to write a free-text response if their reason for reporting was not listed as an option in the questionnaire or if they had additional reasons. A total of 16 participants responded to the free text fields regarding the reasons that motivated them to report ADRs. Commonly reported reasons for deciding to report the ADR were because they wanted to get an alternative medicine (*n* = 7) and contribute to the safety of medicine (*n* = 3).

### Consumers’ reasons for not reporting ADRs

**Fig. 2:**
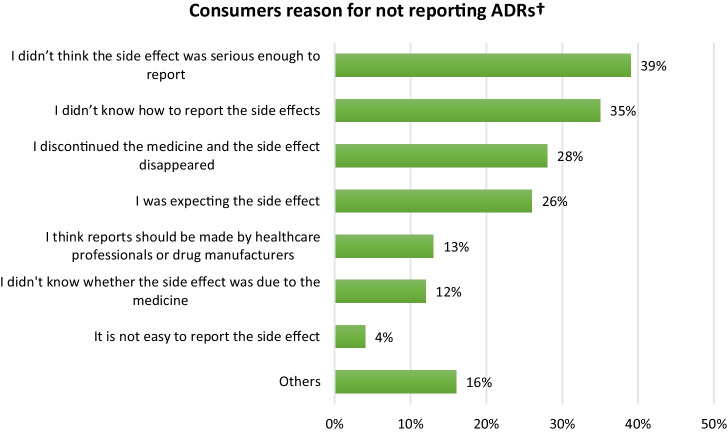
Reasons for consumers who experienced ADRs but did not report them (*N* = 254). †Respondents have the option to choose multiple responses

The 254 respondents who had experienced an ADR but did not report it were asked to select from a list of the factors that influenced their decision. The main reasons for not reporting ADRs were that they did not think the ADR was serious enough to report (39%), and they did not know how to report ADRs (35%) (Fig. 2). Participants were asked to provide a free-text response if their reasons for not reporting ADRs were not included in the questionnaire, or if they had additional reasons. A total of 41 respondents stated additional or other reasons, with commonly reported reasons as follows: they assumed their healthcare professional would report it (*n* = 21); they did not know they could report ADRs (*n* = 5); they experienced a known ADR (*n* = 5); and they experienced the ADR a long time ago (*n* = 3).

### Chi-squared test between ADR reporting and respondent characteristics

Consumers who had experienced ADRs (*n* = 405) were categorized as those who had reported or not reported ADRs. Following the exclusion of consumers who were not sure whether they reported ADRs (*n* = 6), chi-squared tests were conducted for the remaining 399 respondents to determine whether there was a difference between respondents’ characteristics and whether or not they reported ADRs. Results showed that awareness of the blue card adverse reaction reporting form has shown a significant difference between consumers who had and had not reported ADRs (Table 4). As indicated in Table 4, 22% of the respondents who had reported an ADR were aware of the blue card ADR reporting form whereas only 8% of respondents who had not reported an ADR were aware of the form (*p* < 0.001).

**Table 4:** Characteristics of the respondents by ADR reporting (*N* = 399)

**Variables**		**Reported ADR**		***p*-value**
		**Yes, *n* = 145**	**No, *n* = 254**	
Gender	Man	27 (18.6%)	44 (17.3%)	0.803
	Woman	105 (72.4%)	191 (75.2%)	
	Others or no response	13 (9.0%)	19 (7.5%)	
Age	18–24	3 (2.1%)	20 (7.9%)	0.165
	25–34	16 (11.0%)	33 (13.0%)	
	35–44	20 (13.8%)	26 (10.2%)	
	45–54	32 (22.1%)	38 (15.0%)	
	55–64	22 (15.2%)	46 (18.1%)	
	65–74	32 (22.1%)	57 (22.4%)	
	75 or older	9 (6.2%)	19 (7.5%)	
	No response	11 (7.6%)	15 (5.9%)	
Highest level of education	Secondary school	18 (12.4%)	48 (18.9%)	0.080
	VET certificates, diploma, associate diploma, associate degree, or graduate diploma	14 (9.7%)	10 (3.9%)	
	Bachelor’s degree	46 (31.7%)	91 (35.8%)	
	Postgraduate degree	52 (35.9%)	81 (31.9%)	
	Others or no response	15 (10.3%)	24 (9.4%)	
The average number of medicines taken	1–4	66 (45.5%)	153 (60.2%)	0.011
	≥ 5	62 (42.8%)	85 (33.5%)	
	No response	17 (11.7%)	16 (6.3%)	
Start taking a new medicine for the first time	Yes	82 (56.6%)	126 (49.6%)	0.182
	No	63 (43.4%)	128 (50.4%)	
Used digital tools for healthcare activities	Yes	138 (95.2%)	240 (94.5%)	0.136
	No	4 (2.8%)	13 (5.1%)	
	No response	3 (2.1%)	1 (0.4%)	
Level of experience with using a smartphone	Limited	8 (5.5%)	6 (2.4%)	0.561
	Moderate	25 (17.2%)	43 (16.9%)	
	Advanced	108 (74.5%)	198 (78.0%)	
	Don’t use a smartphone	1 (0.7%)	2 (0.8%)	
	No response	3 (2.1%)	5 (2.0%)	
Awareness of blue card adverse reaction reporting form	Yes	32 (22.1%)	19 (7.5%)	< 0.001
	No	111 (76.6%)	229 (90.2%)	
	Not sure	2 (1.4%)	6 (2.4%)	
Awareness of the black triangle scheme	Yes	20 (13.8%)	16 (6.3%)	0.037
	No	122 (84.1%)	234 (92.1%)	
	Not sure	3 (2.1%)	4 (1.6%)	

## Discussion

This study assessed consumers’ knowledge and experience of ADR reporting and their use of digital tools for reporting ADRs in Australia. More than half (58%) of the Australian consumers who responded to our survey knew that they could report ADRs; 35% knew how to report ADRs and 15% reported using digital tools. The main reason for not using a digital tool to report the ADR was that they were not aware that they could report using digital tools.

The proportion of Australian consumers in our study who said they knew that they could report ADRs (58%) is much higher than reported in other studies conducted in Australia [23], Europe [22, 33], and Asia [18, 29] that reported 9 to 17% of consumers were aware that they could report ADRs. All but one of these earlier studies were conducted prior to the start of the COVID-19 pandemic [18, 22, 23, 33], and one was conducted in March and April of 2020 at the beginning of the pandemic [29]. The higher rate of awareness of ADR reporting by consumers in our study may be associated with widespread advertising of the importance of COVID-19 vaccine ADR reporting on social media and through flyers at vaccine clinics [34, 35] and an associated heightened awareness among consumers of the importance of reporting ADRs. A study conducted in Lithuania in the aftermath of the COVID-19 pandemic found similar results to our study, with 64% of consumers aware that they could report ADRs [36] which provides some support for this hypothesis. However, a 2015 study from Portugal undertaken before the pandemic found that 53% of consumers were aware that they could report ADRs [37] indicating that increased awareness post-COVID is not the only contributing factor. The difference could also be related to differences in the wording of the survey questions — our survey asked whether the consumers knew that they could report ADRs to either the government or healthcare professionals, whereas the previous studies asked consumers whether they knew they could report ADRs to the government. Another possible explanation for the high proportion of consumers in our study who knew about ADR reporting was due to our sampling method, whereby people who were more knowledgeable or interested in the topic of ADR reporting may have been more likely to complete the survey. Additionally, Australia has had a long-standing consumer ADR reporting line and a quality use of medicines program with strong consumer engagement that may have enhanced consumer awareness of ADR reporting in this country [38–40].

In Australia, consumers have the option to report ADRs over the phone, online via the TGA website, or by email. Despite more than half of the consumers in our study being aware that they could report ADRs, only one-third were aware of how to report them. A similar finding was reported in a study conducted in the UK in 2019, where only 18% of respondents knew how to report an ADR [41]. Additionally, only 13% of respondents were aware of the blue card adverse reaction reporting form, which is an ADR reporting form in Australia similar to the yellow card reporting form used in the UK [42]. The blue card was previously distributed to healthcare professionals in printed form, but it is now only accessible online on the TGA website [10, 43]. As shown by our study, most respondents were not aware of this option.

In total, 11% of respondents were aware of the black triangle scheme. In Australia, the black triangle scheme was introduced in 2018 on the packaging of newly approved medicines [44]. The purpose of this scheme is to alert healthcare professionals and consumers to the potential for ADRs associated with these new medicines, serving as a prompt to report ADRs [44]. This scheme is similar to the black triangle scheme operated in the European Union and the UK [45]. A survey conducted in European Union countries reported that only 30% of patients were aware of the black triangle scheme [46]. These findings highlight the need for increased efforts to raise awareness among consumers about the purpose and meaning of the black triangle scheme.

Eighty percent of the consumers who responded to our survey who had reported an ADR said that they reported it to their healthcare professionals, with 69% reporting in person. Of the 100 respondents who reported an ADR in person, 78% had an advanced level of experience with smartphones. This suggests that while individuals use digital tools for health-related activities, they mainly communicate face-to-face with their healthcare professionals for ADR reporting instead of using digital tools. Of the consumers in our study who had reported an ADR, only 15% reported their ADR using digital tools. The lack of use of digital tools was primarily because the consumers were unaware that such tools were available (58%), while 22% had concerns about data privacy and security, and 9% found the online reporting of ADRs confusing. Addressing consumers’ concerns regarding data privacy and security of using digital tools and increasing the awareness of the need to report ADRs to the TGA may increase online ADR reporting by consumers. This may be achieved through various strategies such as providing clear and transparent information about how patient data will be collected, stored, and used, clearly communicating the purpose of collecting data and the measures in place to ensure privacy [47].

In our study, the main reasons cited by consumers who reported ADRs were because they were worried about their own situation and because they experienced serious ADRs. This finding aligns with findings of studies conducted in the Netherlands [30, 31], Portugal [37], and European Union countries [46], which reported that consumers’ main reasons for reporting ADRs were to share their experiences, the severity of the reaction, worries about their own situation, and because the ADR influenced their daily activities. Our study found that the main reasons for consumers not reporting ADRs were that they did not think the ADR was serious enough to report, and they did not know how to report ADRs. Consistent with this finding, previous studies have reported that consumers’ main reasons for not reporting ADRs were that the ADRs were not serious enough to report, that there was a lack of knowledge about what happens with the report, and that there was a lack of awareness about how to report ADRs [31, 36, 48]. A systematic review of studies reported that poor patient awareness of available reporting systems was the primary barrier to reporting ADRs [16]. These findings indicate a lack of understanding among consumers regarding the importance of reporting all types of ADRs and a lack of knowledge about reporting methods. These findings underscore the need to enhance consumer awareness about the reporting of all types of ADRs and reporting methods through various media channels and with the assistance of healthcare professionals.

While this study attempted to include a sample of study participants from all states of Australia, there are certain limitations to be considered. Specifically, the study utilized a convenience sampling method, which may have resulted in selection bias if only those interested in the study topic participated. The study was conducted online, and thus, individuals who do not use electronic devices may not have been adequately represented. Although paper-based survey forms were offered as an alternative to online participation, no paper-based surveys were returned to us. Additionally, as the study was conducted online some results are likely overestimated. For example, the percentage of people able to report ADRs online is probably higher than the true figure, because our sampling method targeted people who are already familiar with using online platforms. In comparison with the general population of Australia, where 51% of people aged between 20 and 74 years old have a bachelor’s degree or higher [49, 50], a slightly larger proportion of participants in our study held bachelor’s degrees or higher (64% of study participants). Furthermore, a higher proportion of women (68% vs 50%) and individuals aged 65 years or older (28% vs 17%) were present in our study sample compared to the general Australian population [51–53]. Similarly, a higher proportion of participants from South Australia (15% in our survey responses vs 7% in the general population) and a lower proportion from Western Australia (2% in our survey responses vs 10% in the general population) participated in our study, while the proportion of respondents in other states generally matched the proportion of the general population living in these states [53]. These factors may limit the generalizability of the study. While we conducted univariate analyses of the characteristics of respondents who reported and did not report ADRs, a multivariate prediction model of the factors that predict the likelihood of reporting ADRs would be useful and could be the subject of future research in this area.

## Conclusion

Over half of the consumers who responded to our survey knew that they could report ADRs; however, their knowledge of ADR reporting methods, using digital tools for ADR reporting, and practices of ADR reporting were low. Addressing awareness of digital tools for reporting ADRs, privacy and security concerns are crucial in order to encourage the use of digital tools for ADR reporting, as a large proportion of respondents described these as barriers to ADR reporting using digital tools. Future research should focus on evaluating consumer interest in using digital tools for ADR reporting and assessing the impact of digital tools on the rate and quality of ADR reporting.

## Supplementary Information

Below is the link to the electronic supplementary material.

Supplementary file1 (DOCX 56 KB)

## Data Availability

The data supporting this study's findings are available on request from the corresponding author.
