# Supplementary material for: Consumers’ knowledge and experiences of adverse drug reaction reporting in Australia: a national survey
Source: Eur J Clin Pharmacol. 2024 Jul 12;80(10):1543–54. doi: 10.1007/s00228-024-03729-y (PMC11393011; doi:10.1007/s00228-024-03729-y)
Supplement: Supplementary file 1 — Supplementary file1 (DOCX 56 KB) [file 228_2024_3729_MOESM1_ESM.docx]

**Supplementary Material 1: Survey Questionnaire**

**The following terms are used in the survey, and they are defined as follows:**

**Side effects** are any unintended effects related to the medicines you take.

**Therapeutic Goods Administration (TGA)** is the medicine regulatory agency of the Australian Government.

**Healthcare professionals** include doctors, pharmacists, nurses, midwives, dentists, physiotherapists and other registered health professionals involved in patient care.

**Medicines** include medicines or preparations prescribed to you by a doctor or purchased over the counter from the pharmacy or supermarket.

1. **General questions on experience with medicine use.**

**This section seeks to understand your history of medicine use over the past six months.**

1. Please select the option that best describes your experience with medicine use in the past 6 months:

I have used one or more medicine(s) in the past 6 months

I am unsure if I have taken medicine in the past 6 months *(Thank you for your time. This survey is for people who have taken medicine in the past 6 months)*

I have not taken any medicine in the past 6 months *(Thank you for your time. This survey is for people who have taken medicine in the past 6 months)*

1. What medicines have you taken in the past 6 months? (Please select all options that apply)

Medicines prescribed to me by a healthcare professional

Non-prescription or over-the-counter medicines

Other, please specify _______________________________________________________ ________________________________________________________________

1. In the past 6 months, how many medicines did you use (on average) each day?__________________________________________________________________ ___________________________________________________________ (free text)
2. Did you start taking a new medicine for the first time in the past 6 months?

Yes

No

Not sure

1. **Knowledge about medicine side effect reporting.**

**This section** **seeks to understand what consumers know about the reporting of side effects from medicines.**

1. Members of the public, healthcare professionals and the companies that sell medicines can all report side effects from medicines. Side effects due to medicines can be reported by the consumers to the Therapeutic Goods Administration, State or territory government health departments, or healthcare professionals.

Did you know that you can report side effects if you suspect they are due to a medicine you are taking before reading about it here?

Yes

No *(go to #7)*

1. Please select the organisations you are aware of where side effects can be reported to. (Please select all options that apply) *(logic: only for people who answer “Yes” for #5)*

the Therapeutic Goods Administration

the State or Territory government health department

Healthcare professionals

I am not sure where I can report side effects from medicines

Other (please specify)___________________________________________________ ___________________________________________________________________

1. If you wanted to report a side effect due to a medicine to the Therapeutic Goods Administration, state or territory government health department or healthcare professionals, would you know how to go about it?

Yes

No *(go to #9)*

Not sure *(go to #9)*

1. Which side effect reporting method(s) are you aware of? (Please select all options that apply) *(logic: only for people who answer “Yes” for #7)*

Phone call

Email

Online via the Therapeutic Goods Administration a website

Post

Other (please specify)_____________________________________________

1. The blue card adverse reaction reporting form developed by the Therapeutic Goods Administration is used to report any suspected side effects from vaccines or medicines. It can be filled out either electronically or by printing a hard copy and submitting it either through email or postal mail.

Had you heard about the blue card adverse reaction (side effect) reporting form before reading about it here?

Yes

No

Not sure

1. The black triangle scheme is represented by a black triangle symbol (
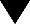
) on medicinal product information and consumer medicine information documents and is intended to remind healthcare professionals and consumers to report suspected side effects related to medicines.

Had you heard about the black triangle scheme before reading about it here?

Yes

No

Not sure

1. **Practices of medicine side effect reporting.**

**This section collects information regarding your experiences in reporting of side effects from medicine.**

1. Have you ever experienced a side effect from any medicine?

Yes

No *(go to #17)*

Not sure *(go to #17)*

1. Have you ever reported a medicine side effect to the Therapeutic Goods Administration, state or territory government health department or healthcare professionals? *(logic: only for people who answer “Yes” for #11)*

Yes

No *(go to #15)*

Not sure *(go to #17)*

1. Who did you report the medicine’s side effects to? If you have reported more than one time, tell us about the most recent time. (Please select all options that apply) *(logic: only for people who answer “Yes” for #12)*

the Therapeutic Goods Administration

the State or Territory government health department

a healthcare professional

other (please specify)______________________________________________________ __________________________________________________

1. How did you report the most recent side effect you experienced? (Please select all options that apply) *(logic: only for people who answer “Yes” for #12)*

Via Phone conversation

Via Email

Online form via the Therapeutic Goods Administration website

Via Post

In person

Other (please specify)_____________________________________________________ _____________________________________________________

1. Why did you decide to report the medicine’s side effects? (Please select all options that apply) *(logic: only for people who answer “Yes” for #12)*

I don’t want other people to experience the same side effect

I want to know more about what I experienced

I experienced a serious side effect/s

I was told by a healthcare professional to report the side effects

I wanted to share my experiences

I was worried about my own situation

I was angry about what happened

Other (please specify): _____________________________________________________ ________________________________________________________________________________________________________________________________________________

1. Why did you choose not to report the medicine’s side effects? (Please select all options that apply) *(logic: only for people who answer “No” for #12)*

I didn’t think the side effect was serious enough to report

I discontinued the medicine and the side effect disappeared

I was expecting the side effect

It is not easy to report the side effect

I didn’t know how to report the side effects

I didn't know whether the side effect was due to the medicine

I think reports should be made by healthcare professionals or drug manufacturers

Other (please specify): ____________________________________________________

________________________________________________________________________________________________________________________________________________

1. **Views on the use of digital tools (for example a mobile app, web app or website) for medicine side effect reporting.**

**This section aims to understand your views on the** **use** **of digital tools for reporting a medicine side effects.**

**The following terms are used in this section, and they are defined as follows:**

**A digital tool** is a mobile app, web app or website that generates data and allows you to create a medicine side effect report on a portable device such as a smartphone, tablet or laptop.

**Electronic devices** are smartphones, tablets or laptops.

1. How would you rate your level of experience with using a smartphone?

Limited (I use it to make a phone call and send text messages only)

Moderate (I use it for online searches such as on Google and Safari)

Advanced (I use it to make e-payments, book appointments using apps etc)

I don’t use a smartphone

1. Have you ever used digital tools for healthcare activities, for example, making or confirming doctor’s appointments?

Yes

No

Not sure

1. Have you ever used digital tools for reporting medicine side effects? *(logic: only for people who answer “Yes” for #12)*

Yes

No

Not sure

If yes, please write the digital tool you used if you remember the name *(then go to #21)* ______________________________________________________________________ ________________________________________________________________

1. Why didn’t you use a digital tool for reporting the side effects? (Please select all options that apply) *(logic: only for people who answer “No” for #19)*

I wasn’t aware that I could report using digital tools

I find online reporting digital tools confusing

I have data privacy concerns

I have security concerns

I didn’t have an electronic device

Other reasons (please specify) ____________________________________________ _____________________________________________________________________

1. If a digital tool was developed for reporting medicine side effects, how important are the following features to you?

| S.No | Features | Not at all important | Somewhat important | Important | Very important | Don’t know |
| --- | --- | --- | --- | --- | --- | --- |
|  | It enables you to share the report you submit about the side effect with your healthcare professionals |  |  |  |  |  |
|  | It allows you to upload documents or evidence of the side effect (for example a photo of your symptoms like a skin rash, a photo of the medicine you were taking) |  |  |  |  |  |
|  | It allows you to complete a report offline and submit it later when you have internet access |  |  |  |  |  |
|  | It provides a drop-down menu with tick-box options to select side effects and medicines |  |  |  |  |  |
|  | It provides a free text space for describing the side effects in your own words |  |  |  |  |  |
|  | It enables you to receive information on the safety of medicine |  |  |  |  |  |
|  | It enables you to receive a summary of side effects previously reported to the regulatory agency |  |  |  |  |  |
|  | It enables you to receive a message acknowledging your report submission |  |  |  |  |  |

Please write here if there are other features that you would like to see in a digital tool for reporting medicine side effects that are not mentioned above. _____________________________________________________________________________________________________________________________________________________________________________________________________________________________________________________________________________________________________

1. This section aims to identify factors that might affect your intention to use digital tools to report medicine side effects. Please indicate to what extent you agree or disagree with the following statement.

|  | | Strongly disagree | Disagree | Neutral | Agree | Strongly agree |
| --- | --- | --- | --- | --- | --- | --- |
| 1. | A digital tool to report medicine side effects seems to be useful to me. |  |  |  |  |  |
| 2. | I think using digital tools will enable me to report a medicine side effects quicker than using other methods. |  |  |  |  |  |
| 3. | It would be easy for me to use digital tools to report side effects from medicines. |  |  |  |  |  |
| 4. | Using digital tools to report side effects from medicines is a good idea. |  |  |  |  |  |
| 5. | I would be more likely to report a medicine side effect using a digital tool if my healthcare professional encouraged me to do so. |  |  |  |  |  |
| 6. | I would be more likely to report a medication side effect using a digital tool if someone showed me how. |  |  |  |  |  |
| 7. | I have the necessary knowledge to use electronic devices for reporting medicine side effects. |  |  |  |  |  |
| 8. | If I need help to use a digital tool to report a medicine side effect, I know people who would be able to assist me. |  |  |  |  |  |
| 9. | I would intend to use digital tools for reporting a medicine side effects in the future. |  |  |  |  |  |

1. Please rank the following reporting methods based on how comfortable you feel using them. Rank from 1=very comfortable to 4=least comfortable.

Paper-based form

Phone conversation

Website or Web app

Mobile app

1. **Consumers’ socio-demographic characteristics.**

**This section is intended to collect information about you.**

1. What gender do you identify as?

Man

Woman

I identify my gender as:_____________________________________

Prefer not to say

1. What is your age group?

18-24

25-34

35-44

45-54

55-64

65-74

75-84

85 or older

1. What is your highest level of education?

Primary school

Secondary school

Bachelor’s degree

Postgraduate degree

Prefer not to say

Other (please describe):_______________________________________________

1. What state do you currently live in?

New South Wales

Queensland

Northern Territory

Western Australia

South Australia

Victoria

The Australian Capital Territory

Tasmania
